# The effects of Baduanjin exercise vs. brisk walking on physical fitness and cognition in middle-aged patients with schizophrenia: A randomized controlled trial

**DOI:** 10.3389/fpsyt.2022.983994

**Published:** 2022-10-05

**Authors:** Chyi-Rong Chen, Yu-Chi Huang, Yi-Wen Lee, Hui-Hsien Hsieh, Yi-Chen Lee, Keh-chung Lin

**Affiliations:** ^1^Department of Psychiatry, Kaohsiung Chang Gung Memorial Hospital and Chang Gung University College of Medicine, Kaohsiung, Taiwan; ^2^School of Occupational Therapy, College of Medicine, National Taiwan University, Taipei, Taiwan; ^3^Department of Nursing, Kaohsiung Chang Gung Memorial Hospital and Chang Gung University College of Medicine, Kaohsiung, Taiwan; ^4^Division of Occupational Therapy, Department of Physical Medicine and Rehabilitation, National Taiwan University Hospital, Taipei, Taiwan

**Keywords:** schizophrenia, Baduanjin, brisk walking, mind-body exercises, physical fitness, cognition

## Abstract

**Objective:**

Patients with schizophrenia have deficits in physical and cognitive function that may become salient in their middle ages. These deficits need active intervention to prevent functional decline. Baduanjin and brisk walking show promise as interventions in patients with schizophrenia. This study investigated the effects of Baduanjin exercise vs. brisk walking in middle-aged patients with schizophrenia.

**Methods:**

In this single-blind, 2-arm, parallel, randomized controlled trial, 48 participants aged older than 40 years were enrolled and assigned to the intervention group (Baduanjin) or the control group (brisk walking). The training of both groups took place twice a week, 60 min per session, for 12 weeks. The participants were evaluated with physical, cognitive, and functional outcomes at baseline, postintervention, and 4 weeks after the intervention.

**Results:**

The results of the study demonstrated significant time effects in walking distance (*p* = 0.035, η^2^ = 0.094) and lower extremity strength (*p* = 0.006, η^2^ = 0.152). *Post-hoc* analysis revealed both groups had significant improvement in changes from baseline to the postintervention assessment (*p*s < 0.05) and follow-up (*p*s < 0.05). The results demonstrated a significant group-by-time interaction in change scores of global cognition (*F* = 7.01, *p* = 0.011, η^2^ = 0.133). *Post-hoc* analysis revealed a significant improvement in the Baduanjin group from baseline to postintervention (*p* = 0.021), but the improvements were not maintained at the follow-up assessment (*p* = 0.070). The results also demonstrated significant group effects in balance function (*p* < 0.001, η^2^ = 0.283), motor dual-task performance (*p* = 0.026, η^2^ = 0.103), and cognitive dual-task performance (*p* < 0.001, η^2^ = 0.307). *Post-hoc* analysis revealed that the Baduanjin group improved more than the brisk walking group in the above outcomes (*p*s < 0.05).

**Conclusion:**

This study demonstrated the differential effects of Baduanjin exercise and brisk walking in middle-aged patients with schizophrenia. Baduanjin might be a beneficial regimen for improving physical and cognitive function in this population. Further research with a larger sample is warranted.

**Clinical trial registration:**

[ClinicalTrials.gov], identifier [202000817B0C602].

## Introduction

Schizophrenia is a serious mental illness (1). Evidence shows that individuals with schizophrenia may display accelerated aging in physiological functions such as increased oxidative stress and inflammation (2). Individuals with schizophrenia usually have sedentary lifestyles, poorer physical health and functional aerobic capacity, and shorter life expectancy than the general population (3, 4). In addition to physical health problems, cognitive impairment is a core deficit in schizophrenia and has been identified as a key predictor of the patient’s ability to perform functional activities (5, 6). Research has discovered that patients with schizophrenia show an accelerated rate of white matter loss (7) and significant deficits in executive function (6) at ages 35–40 years. Patients with schizophrenia also display deficits in cognitive and motor dual-tasks and have difficulties executing multiple tasks at the same time (8). Poorer dual-task performance and psychomotor dysfunctions are associated with deterioration of social, clinical, and daily functions in schizophrenia (8, 9).

Antipsychotic medication was shown to be effective in alleviating positive symptoms (e.g., hallucinations and thought disorders) (10); however, medications may not effectively improve negative symptoms (e.g., flat affect and social withdrawal) (11) and cognitive function (12, 13). In addition, antipsychotic medication may be associated with adverse effects and increased risks of metabolic syndromes (10). Physical exercise is a potentially adjunctive treatment to medications (14). Clinical research has shown that exercise improves psychiatric symptoms along with physical and mental health in patients with schizophrenia (15, 16). A meta-analysis found that higher doses of aerobic exercise had a small-to-medium effect on global cognition in schizophrenia (16).

However, patients with schizophrenia usually encounter certain barriers to participation in physical activity due to the illness, such as fatigue, poor motor control, and poor coordination (17). One study estimated that approximately one-third of patients with schizophrenia discontinued the exercise regimen during the intervention (15). Another study found that older veterans with severe mental illness were more likely to have arthritis and cardiopulmonary conditions that impede physical activity (18).

Mindful exercises, such as Baduanjin, Qigong, and Tai Chi, place an emphasis on symmetrical physical posture, internal awareness, deep breathing, and meditation to improve wellness (19, 20). These exercises, which are commonly practiced in the Asian culture, require coordinated mind and body interactions in a harmonious manner (21). Plausible mechanisms for mindful exercise may involve several perspectives. First, mindful exercises, such as Baduanjin and Tai Chi, are light-to-moderate aerobic exercise (22, 23). The biomechanical, physiological, and psychological changes due to aerobic exercise in patients with schizophrenia might be attained through mindful exercises.

Second, mindful exercises require participants to focus on the coordination of movements and regulation of Qi (energy)/breath simultaneously (24, 25), which might be cognitively demanding. This demand for practicing mindful exercises may have a beneficial effect on cognitive function beyond conventional unimodal exercise (25).

Third, the emphasis of the integration of breathing into rhythmic movements might lead to downward regulation of the hypothalamic-pituitary-adrenal axis function. This downward regulation may subsequently ameliorate stress and depression and improve cardiovascular function (26).

Previous research showed that Tai Chi might be too complicated for patients with schizophrenia to learn, resulting in difficulty for them to practice independently (27). Baduanjin is an alternative less complex exercise than Tai Chi (21). A recent review by Vamcampfort et al. indicated that meditation-based mind-body exercises might be used to complement psychotropic medication. However, their work pointed out the lack of rigorous randomized clinical trials exploring the effects of Qigong, such as Baduanjin, in psychotic disorders (28). Compared with research on Tai Chi practice in the elderly or in patients with mental illness, clinical study of Baduanjin has been scarce. A pretest and posttest study by Chen et al. found that after 16 guided practice sessions, patients with severe mental illness were able to practice Baduanjin independently at home for 8 weeks with the help of short message reminders (24). The patients were engaged for 80% of the time expected to be engaged in practice. The study results showed improvement in balance, cognition, and quality of life from the pre- to post-treatment assessment (24). A recent randomized controlled trial demonstrated that Baduanjin practice improved logical memory in patients with chronic schizophrenia immediately after treatment (29). However, the study did not include a follow-up assessment, and the maintenance effect of Baduanjin practice after the intervention remains unclear.

As a popular aerobic exercise, walking might be an alternative exercise appropriate for implementation in patients with schizophrenia (30). A systematic review showed that walking might have a small effect on reductions of body max index in the short-term among patients with schizophrenia (30). Compared with usual care, recent studies of walking showed benefits in quality of life in schizophrenia (31). Another study found that only moderate intensity of walking was effective in improving processing speed (32). The requirement of physical effort to accomplish moderate intensity of walking might be difficult for middle-aged patients with schizophrenia. The physical effort required for exercise when implementing walking interventions in this population needs to be considered.

Despite of the relevance of Baduanjin and walking exercise for individuals with schizophrenia, comparative efficacy research of these interventions in middle-aged adults with schizophrenia is lacking. The purpose of this study was to examine the efficacy of Baduanjin exercise vs. brisk walking on physical fitness, cognitive function, and dual-task performance in middle-aged patients with schizophrenia. We hypothesized that Baduanjin training and brisk walking might have differential effects on physical fitness, cognitive function, and dual-task performance for middle-aged adults with schizophrenia.

## Materials and methods

### Study design

**FIGURE 1:**
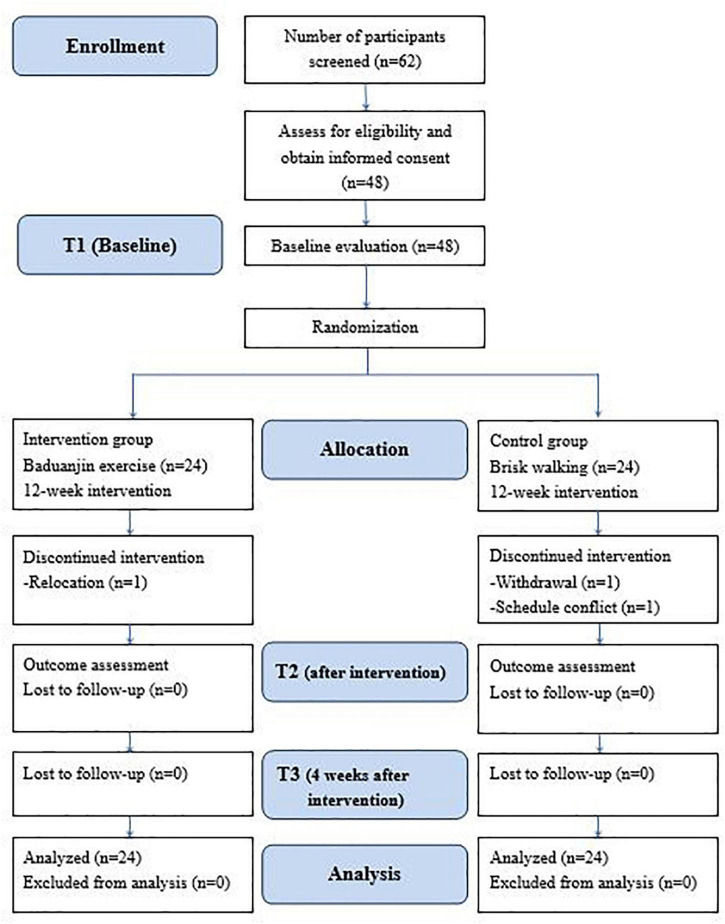
Flow diagram of the study.

This study was a single-blind, 2-armed, randomized, parallel controlled design in which 48 participants were allocated to the intervention group (Baduanjin exercise) or the control group (brisk walking). All participants continued their respective intervention for 12 weeks. Outcome evaluations were conducted at baseline, postintervention, and 4 weeks after the intervention. All participants were informed of the study purpose, and their consent was obtained before enrollment. The flow chart of the study is depicted in Figure 1.

This study was conducted at the day care center of the Chang Gung Memorial Hospital Department of Psychiatry in Taiwan. The study was approved by the Institutional Review Board (Number: 202000817B0C602) and was registered on ClinicalTrials.gov (Identifier: NCT04430309).

### Participants

Patients with schizophrenia at the age of 40 years or older were recruited. Middle-aged participants were recruited for the study because this population may have significant deficits in physical and cognitive function (6), and active intervention is warranted to prevent functional decline that may affect their participation in daily life.

#### Inclusion criteria

Eligibility criteria for inclusion were participants: (1) with a diagnosis of schizophrenia according to the *Diagnostic and Statistical Manual of Mental Disorders*-5 (33); (2) who were 40 years or older; and (3) in stable mental status without a shift in the dosage of medication for at least 1 month. The dosage of antipsychotics was retrieved from medical records. The medication was transformed to chlorpromazine equivalence according to the work by Leucht et al. (34).

#### Exclusion criteria

Participants were excluded if they: (1) had serious physical conditions, such as cardiovascular disease, musculoskeletal disease, or cardiopulmonary disease; (2) had a visual or auditory impairment that may preclude completion of the assessment; (3) required full-time hospitalization; (4) had severe withdrawal or profound intellectual disability; or (5) were a participant in another clinical trial at the time of enrollment to this present study.

### Randomization and blinding

After the baseline assessment, participants were assigned randomly to the experimental group or the control group with a 1:1 ratio based on random tables generated by an independent research assistant with the web-based tool.^1^ The research assistant was not involved with participant recruitment, assessment, or treatment intervention. The outcome assessor was blinded to the group assignment, and participants were reminded not to discuss their intervention with the assessor.

### Intervention

#### Experimental group

Participants in the experimental group received 12 weeks of Baduanjin exercise. Besides the experimental intervention, all routine medical and psychosocial treatments were continued as usual. Baduanjin exercise was conducted for 60 min per session, 2 sessions per week, for a total of 24 sessions in 12 weeks. Each session began with a 10-min warm-up consisting of a series of range of motion activities and deep breathing exercise. After the exercise, there was a 10-min cool down session that included short discussions about the participants’ feelings, perceptions, and thoughts about the practice.

**FIGURE 2:**
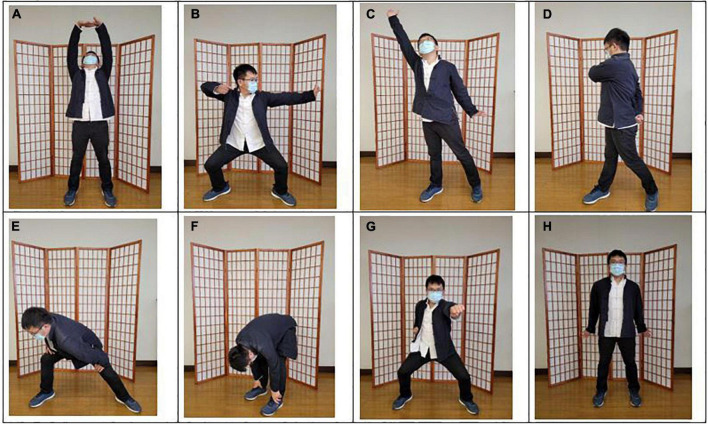
The postures of Baduanjin exercise. **(A)** Both hands holding up the heavens; **(B)** drawing the bow to shoot the eagle; **(C)** separating the heaven and earth; **(D)** wise owl gazing backwards; **(E)** swaying the head and shaking the tail; **(F)** both hands holding the feet to strengthen the waist; **(G)** clenching the fists and glaring fiercely; **(H)** bouncing on the toes.

Baduanjin exercise consists of eight postures, with emphasis on different body parts. The exercise is commonly practiced as follows: (a) both hands holding up the heavens, (b) drawing the bow to shoot the eagle, (c) separating the heaven and earth, (d) wise owl gazing backwards, (e) swaying the head and shaking the tail, (f) both hands holding the feet to strengthen the waist, (g) clenching the fists and glaring fiercely, and (h) bouncing on the toes (Figure 2). We used the Baduanjin practice scheme based on the guidelines of the Baduanjin Qi-Gong manual (35).

Before the intervention, an 8-hour training program was given to the occupational therapists and nurses who supervised Baduanjin exercise, meditative techniques, and breathing exercise. They were instructed to use strategies to enhance compliance, such as demonstration of the postures and guiding questions to elicit thoughts about the movements. Baduanjin exercise was practiced in groups with six to eight participants. The attendance rate of the Baduanjin group was recorded. After each session, a modified 10-point Borg scale was used to record the perceived exertion of the participants (36).

#### Control group

Participants in the control group received brisk walking activity for 60 min per session, 2 sessions per week for 12 weeks, and received their usual medical and psychosocial treatment. The brisk walking activity was supervised by occupational therapists and nurses who were not involved in the Baduanjin group. The attendance rate of the brisk walking group was recorded. After each brisk walking session, participants rated their perceived exertion of physical activity with a modified 10-point Borg scale (36).

### Outcome measures

The researchers collected baseline data, including sex, age, body mass index, education, antipsychotic drug dosage, and age at illness onset. The scores of Clinical Global Impression scale-severity (CGI-S) (37) of the participants were retrieved through medical records. Treatment outcomes in this study included physical function, cognitive function, dual-task performance, and activities of daily living (ADLs).

#### Primary outcomes

##### Six-minute walk test

The six-minute walk test (6MWT) can be easily performed in clinical settings with simple equipment. The participants were asked to walk back and forth within a 25-m distance. The total distance walked was recorded. The longer the distance, the better the cardiovascular function. The test-retest reliability of the 6MWT in individuals with schizophrenia has been established (38).

##### Thirty-second chair stand test

The 30-second chair stand test (30CST) requires participants to sit straight on a chair placed against a wall, with their arms folded across the chest. The participants were asked to rise to a full stand position and then sit down with arms remaining in same position. They were instructed to repeat the movements as quickly as possible in the 30-s period. The repetitions were recorded, and a higher score indicates better lower extremity muscular strength (39).

#### Secondary outcomes

##### Montreal cognitive assessment

The Montreal cognitive assessment (MoCA) is a 30-point test for evaluation of global cognitive function. The test examines visuospatial processing and executive function, naming, immediate and delayed recall, concentration, digit forward and backward span, serial counting, language, abstract thinking, and orientation. A higher score indicates better global cognitive function. The MoCA has been shown to be sensitive in detecting cognitive impairment in schizophrenia (40). The MoCA scores provided information about global cognitive functions in patients with schizophrenia (41). It has been used as an outcome measure of cognition in patients with schizophrenia in a recent trial (42).

##### Trail making test-part A and B

The trail making test-part A (TMT-A) examines visual attention and speed of processing (43), and trail making test-part B (TMT-B) measures cognitive flexibility and task switching (44). In the TMT-A, participants were asked to connect the numbers from 1 to 25 in a sequential order within one consecutive line. The TMT-B required participants to alternately link numbers and characters that are scattered on a page in a sequential manner. The numbers include 1–13 and the characters include 12 Chinese zodiac signs. The time required to complete the task was recorded to indicate the level of cognitive function.

##### Logical memory

The Wechsler Memory Scale Logical Memory subtest was used to evaluate memory function (45). The assessor verbally introduced a short story two times. Study participants were instructed to listen to the story, remember the content, and then recall it 30 min later. Participants were asked to repeat details of each story as precisely as possible. The Logical Memory scores that include immediate and delayed recall scores were recorded.

##### Timed up-and-go test

The timed up-and-go (TUG) test evaluates functional mobility, agility, and balance (46). Participants were asked to stand up from a sitting position, walk 3 m, turn around, walk back, and sit down. The time required to complete the task was recorded by a stopwatch. The TUG test has been shown to be a reliable test in older adults (47).

##### Dual-task performance

The dual-task performance was evaluated by two types of dual-task of the TUG test. The motor dual-task of TUG (TUG-DTm) required the participants to carry a glass of water filled to 3 cm from the top of the glass while performing the TUG (48). The participants were instructed to perform the task as quickly as possible. For the cognitive dual-task of TUG (TUG-DTc), participants were asked to complete the TUG while performing serial 3 counting simultaneously (49). A random number from 80 to 99 was selected, and the participants were instructed to count down until the TUG was completed. The time required to complete the TUG-DTm and the TUG-DTc was recorded. The order of the motor and cognitive dual-task conditions was randomized. There was one practice trial and three official trials for each test. The average scores of the official trials were used for the study.

##### Activities of daily living rating scale III

Activities of daily living rating scale III (ADLRS-III), a self-administered, paper-and-pencil test, was used to assess daily life functions. Previous research showed good test-retest reliability of the ADLRS-III in patients with schizophrenia (50). The test comprises 10 domains, including personal hygiene, independence, leisure activity, common graphics, news, financial ability, traffic, communication, and problem adaptation in daily life. Each domain in the ADLRS-III is rated from 0 to 10, with a total score of 100. A higher score indicates better performance in ADL.

### Statistical analysis

The required sample size was estimated based on the effect size estimates reported in a previous study pertinent to the purpose of our study (29). The effect of Baduanjin on logical memory among patients with schizophrenia was medium to large (partial η^2^ = 0.087) (29). Given an 80% level of statistical power and an α level of 0.05, power analysis revealed that the study needed 40 subjects in total. Taking into account the possible drop-out rate at 20% during the study period, 48 participants were recruited. This sample size estimation was made based on the G*Power 3.1.9.4 software (51).

The demographic characteristics of the study sample are described as mean and standard deviation or frequency and percentage, as appropriate. The Shapiro-Wilk test was used to check for normality of distribution. On the basis of an intention-to-treat analysis, continuous variables were analyzed with the independent-sample *t*-test, and categorical variables were analyzed with the χ^2^ test for baseline comparisons between the two groups. Missing data were handled with the last observation carrying forward method.

For the analysis of primary and secondary outcomes, one-way mixed analysis of variance was applied to determine the treatment effects. Statistical significance was set at α = 0.05 on a 2-tailed test, and partial eta squared (η^2^) was calculated to assess the effect size. According to Cohen’s guideline, η^2^ values of 0.01, 0.06, and 0.14 are considered to be small, medium, and large in effect, respectively (52). The *post-hoc* analysis was performed with *t*-tests to account for the possibility of an inflated type I error rate due to multiple testing. The false discovery rate method proposed by Hochberg and Benjamini (53) was applied to adjust for the *p*-values. The statistical analysis was performed using SPSS 25.0 software (IBM Corp., Armonk, NY).

## Results

### Background information of participants

The study recruited 48 patients with schizophrenia. Three participants dropped out during the study. One participant in the Baduanjin group was relocated to another city, one participant in the control group declined to participate in walking, and another dropped out due to a schedule conflict. The study flow is depicted in Figure 1. There were no significant differences between the groups at baseline in demographic variables such as sex, age, body mass index, level of education, marital status, and living condition (Table 1). The participants in both groups were also comparable in clinical variables such as age of onset, duration of illness, times of acute ward hospitalization, daily dosage of antipsychotic medications, and CGI-S scores (Table 1). At baseline, there were no significant differences between the groups in primary and secondary outcomes. No accidents or adverse events occurred during the study period. The average attendance rate was 0.94 (0.06) and the average score of perceived exertion of the participants was 4.96 (0.97).

**TABLE 1:** Demographic and clinical data.

Variable	Baduanjin (*n* = 24) mean ± SD, frequency (%)	Brisk walking (*n* = 24) mean ± SD, frequency (%)	*t*, χ^2^	*P*-value
**Sex**				
Male	12 (50)	12 (50)		
Female	12 (50)	12 (50)	<0.001	>0.999
Age (years)	50.63 (6.11)	50.92 (7.81)	0.144	0.163
Body mass index (kg/cm^2^)	25.73 (4.87)	25.83 (5.01)	0.067	0.947
**Education**				
No formal education	1 (4.2)	0 (0.0)		
Elementary school	1 (4.2)	3 (12.5)		
Junior high school	3 (12.5)	6 (25.0)		
Senior high school	12 (50.0)	10 (41.7)		
University/college	7 (29.1)	5 (20.8)	3.515	0.476
**Marital status**				
Single	19 (79.2)	19 (79.2)		
Married	1 (4.2)	2 (8.3)		
Divorced	4 (16.6)	3 (12.5)	0.476	0.788
**Living condition**				
Living alone	7 (29.1)	5 (20.8)		
Living with family	17 (70.8)	19 (79.2)	0.444	0.505
Onset of illness (years)	23.38 (5.70)	23.71 (6.10)	0.196	0.846
Duration of illness (years)	27.25 (7.75)	27.21 (7.33)	0.019	0.892
Times of acute-ward hospitalization	5.17 (3.46)	4.63 (2.55)	0.617	0.131
Chlorpromazine equivalence (mg/d)	467.77 (224.07)	414.95 (240.67)	0.787	0.746
CGI-S	3.63 (0.65)	3.46 (0.59)	0.933	0.355
6MWT	427.37 (71.11)	447.25 (74.09)	–0.949	0.348
30CST	14.42 (3.59)	12.96 (3.78)	1.371	0.177
TUG	8.46 (1.56)	8.16 (1.99)	0.567	0.573
TUG-DTm	13.28 (3.69)	11.89 (3.39)	1.356	0.182
TUG-DTc	14.95 (4.70)	15.08 (4.53)	–1.04	0.918
MoCA	21.46 (4.09)	22.42 (3.93)	–0.828	0.412
TMT-A	82.03 (33.42)	79.13 (31.58)	0.310	0.758
TMT-B	165.10 (76.53)	152.60 (91.83)	0.512	0.611
LM immediate	16.63 (9.40)	15.79 (7.51)	0.339	0.736
LM delay	8.75 (5.24)	8.67 (5.21)	0.055	0.956
ADL	61.54 (13.11)	64.98 (14.12)	–0.874	0.387

CGI-S, Clinical Global Impression scale-Severity; 6MWT, Six-Minute Walk Test; 30CST, 30-second chair stand test; TUG, Timed Up-and-Go Test; TUG-DTm, motor dual-task Timed Up-and-Go Test; TUG-DTc, cognitive dual-task Timed Up-and-Go Test; MoCA, Montreal Cognitive Assessment; TMT-A, Trail Making Test-Part A; TMT-B, Trail Making Test-Part B; LM, Logical Memory of the Wechsler Memory Scale; ADL, Activities of Daily Living Rating Scale III.

### Interaction effect

There were no significant interactions on walking distance (*F* = 0.62, *p* = 0.805, η^2^ = 0.001), lower extremity strength (*F* = 2.52, *p* = 0.119, η^2^ = 0.052), balance (*F* = 1.30, *p* = 0.260, η^2^ = 0.027), motor dual-task performance (*F* = 0.14, *p* = 0.707, η^2^ = 0.003), cognitive dual-task performance (*F* = 0.15, *p* = 0.699, η^2^ = 0.003), speed of processing (*F* = 1.38, *p* = 0.246, η^2^ = 0.029), cognitive flexibility (*F* = 0.01, *p* = 0.977, η^2^ < 0.001), immediate logical memory (*F* = 0.62, *p* = 0.437, η^2^ = 0.013), delayed logical memory (*F* = 0.03, *p* = 0.860, η^2^ = 0.001), and ADL (*F* = 0.98, *p* = 0.328, η^2^ = 0.021) (Table 2).

**TABLE 2:** Change scores of the outcome measures.

Outcome measures	Baduanjin mean (SD)	Brisk walking mean (SD)	Time (*F*, *P*-value, η^2^)	Group (*F*, *P*-value, η^2^)	Time × Group (*F*, *P*-value, η^2^)
**6MWT**					
T2-T1	25.71 (19.25)	20.85 (20.18)			
T3-T1	21.00 (22.82)	14.23 (16.74)	4.75, 0.035, 0.094	0.60, 0.44, 0.013	0.62, 0.81, 0.001
**30CST**					
T2-T1	1.83 (1.61)	1.13 (0.99)			
T3-T1	1.00 (1.53)	0.83 (1.34)	8.23, 0.006, 0.15	1.46, 0.234, 0.031	2.52, 0.12, 0.052
**TUG**					
T2-T1	−1.01 (0.79)	−0.18 (0.39)			
T3-T1	−0.95 (0.80)	−0.30 (0.55)	0.16, 0.69, 0.003	18.14, <0.001, 0.28	1.30, 0.26, 0.027
**TUG-DTm**					
T2-T1	−1.21 (0.89)	−0.69 (0.90)			
T3-T1	−1.03 (0.92)	−0.45 (0.78)	6.21, 0.016, 0.12	5.30, 0.026, 0.10	0.14, 0.71, 0.003
**TUG−DTc**					
T2-T1	−1.75 (1.22)	−0.28 (1.07)			
T3-T1	−1.67 (1.36)	−0.14 (1.05)	1.89, 0.178, 0.039	20.34, <0.001, 0.31	0.15, 0.70, 0.003
**MoCA**					
T2-T1	0.58 (0.97)	025 (0.85)			
T3-T1	0.29 (0.75)	0.38 (0.92)	1.13, 0.29, 0.024	0.27, 0.61, 0.006	7.01, 0.011, 0.13
**TMT-A**					
T2-T1	−8.82 (9.41)	−5.29 (5.06)			
T3-T1	−8.30 (11.25)	−6.22 (6.51)	0.12, 0.74, 0.002	1.43, 0.24, 0.030	1.38, 0.25, 0.029
**TMT-B**					
T2-T1	−19.49 (17.48)	−12.08 (14.37)			
T3-T1	−18.42 (19.69)	−11.07 (14.82)	1.00, 0.32, 0.021	2.45, 0.12, 0.051	0.01, 0.98, <0.001
**LM-immediate**					
T2-T1	0.92 (1.59)	0.67 (1.20)			
T3-T1	0.54 (1.81)	0.50 (1.38)	4.16, 0.047, 0.083	0.12, 0.73, 0.003	0.62, 0.44, 0.013
**LM-delayed**					
T2-T1	0.88 (1.91)	0.29 (1.73)			
T3-T1	0.75 (1.29)	0.21 (1.96)	0.79, 0.38, 0.017	1.64, 0.21, 0.034	0.03, 0.86, 0.001
**ADL**					
T2-T1	1.69 (0.92)	0.92 (1.93)			
T3-T1	1.77 (3.19)	0.44 (1.92)	0.48, 0.49, 0.01	2.53, 0.12, 0.052	0.98, 0.33, 0.021

T1, Baseline; T2, postintervention; T3, 4 weeks after the intervention; 6MWT, Six-Minute Walk Test; 30CST, 30-second chair stand test; TUG, Timed Up-and-Go Test; TUG-DTm, motor dual-task Timed Up-and-Go Test; TUG-DTc, cognitive dual-task Timed Up-and-Go Test; MoCA, Montreal Cognitive Assessment; TMT-A, Trail Making Test-Part A; TMT-B, Trail Making Test-Part B; LM, Logical Memory of the Wechsler Memory Scale; ADL, Activities of Daily Living Rating Scale III.

There was a significant group-by-time interaction in global cognition (*F* = 7.01, *p* = 0.011, η^2^ = 0.133) (Table 2). *Post-hoc* analysis revealed the Baduanjin group demonstrated significantly better performance at postintervention (*t* = 2.93, *p* = 0.021) but not at follow-up (*t* = 1.90, *p* = 0.070) (Table 3).

**TABLE 3:** *Post-hoc* analysis of the within-group factor.

Outcome measure	Baduanjin	*t*-value	*P*-value^$^	Brisk walking	*t*-value	*P*-value^$^
**6MWT**						
T2-T1	25.71 (19.25)	6.55	<0.001	20.85 (20.18)	5.06	<0.001
T3-T1	21.00 (22.82)	4.51	<0.001	17.11 (20.35)	4.16	<0.001
**30CST**						
T2-T1	1.83 (1.61)	5.59	<0.001	1.13 (0.99)	5.56	<0.001
T3-T1	1.00 (1.53)	3.20	0.004	0.83 (1.34)	3.04	0.009
**TUG-DTm**						
T2-T1	−1.21 (0.89)	–6.67	<0.001	-0.69 (0.90)	–3.78	0.003
T3-T1	−1.03 (0.92)	–5.48	<0.001	-0.45 (0.78)	–2.82	0.015
**MoCA**						
T2-T1	0.58 (0.97)	2.93	0.021	025 (0.85)	1.45	0.185
T3-T1	0.29 (0.75)	1.90	0.070	0.38 (0.92)	1.99	0.177
**LM- immediate**						
T2-T1	0.92 (1.59)	2.83	0.027	0.67 (1.20)	2.71	0.036
T3-T1	0.54 (1.81)	1.44	0.163	0.50 (1.38)	1.77	0.135

^$^p-value was adjusted by false discovery rate method. T1, Baseline; T2, postintervention; T3, 4 weeks after the intervention; 6MWT, Six-Minute Walk Test; 30CST, 30-second chair stand test; TUG-DTm, motor dual-task Timed Up-and-Go Test; MoCA, Montreal Cognitive Assessment; LM, Logical Memory of the Wechsler Memory Scale.

### Intervention effect over time

There were significant changes in walking distance (*F* = 4.75, *p* = 0.035, η^2^ = 0.094), lower extremity strength (*F* = 8.23, *p* = 0.006, η^2^ = 0.152), motor dual-task performance (*F* = 6.21, *p* = 0.016, η^2^ = 0.119), and immediate logical memory (*F* = 4.16, *p* = 0.047, η^2^ = 0.083) after the intervention (Table 2). In the Baduanjin group, *post-hoc* analysis revealed significant changes between baseline and postintervention in walking distance (*t* = 6.55, *p* < 0.001), lower extremity strength (*t* = 5.59, *p* < 0.001), motor dual-task performance (*t* = −6.67, *p* < 0.001), and immediate logical memory (*t* = 2.83, *p* = 0.027). There were significant changes in walking distance (*t* = 4.51, *p* < 0.001), lower extremity strength (*t* = 3.20, *p* = 0.004), and motor dual-task performance (*t* = −5.48, *p* < 0.001) between the baseline and follow-up (Table 3).

The brisk walking group showed significant change from baseline to postintervention in walking distance (*t* = 5.06, *p* < 0.001), lower extremity strength (*t* = 5.56, *p* < 0.001), motor dual-task performance (*t* = −3.78, *p* = 0.003), and immediate logical memory (*t* = 2.71, *p* = 0.036). Performance at the follow-up assessment on walking distance (*t* = 4.16, *p* < 0.001), lower extremity strength (*t* = 3.04, *p* = 0.009), and motor dual-task efficiency (*t* = −2.82, *p* = 0.015) was significantly better than the baseline performance (Table 3).

### Intervention effect between groups

The results showed significant group effects on balance (*F* = 18.14, *p* < 0.001, η^2^ = 0.283), motor dual-task performance (*F* = 5.30, *p* = 0.026, η^2^ = 0.103), and cognitive dual-task performance (*F* = 20.34, *p* < 0.001, η^2^ = 0.307) (Table 2). At the postintervention, *post-hoc* analysis revealed significantly greater improvements in the Baduanjin group than in the control group in balance (*t* = −4.61, *p* < 0.001) and cognitive dual-task performance (*t* = −4.43, *p* < 0.001). A marginally significant difference was found in motor dual-task performance (*t* = −2.01, *p* = 0.051). Similarly, the changes from baseline to the follow-up assessment were significantly greater in the Baduanjin group in measures of balance (*t* = −3.32, *p* = 0.002), cognitive dual-task performance (*t* = −4.36, *p* < 0.001), and motor dual-task performance (*t* = −2.35, *p* = 0.046) (Table 4).

**TABLE 4:** *Post-hoc* analysis of the between-group factor.

Group	Baduanjin	Brisk walking	*t*-value	*P*-value^$^
**TUG**				
T2-T1	−1.01 (0.79)	−0.18 (0.39)	–4.61	<0.001
T3-T1	−0.95 (0.80)	−0.30 (0.55)	–3.32	0.002
**TUG-DTm**				
T2-T1	−1.21 (0.89)	−0.69 (0.90)	–2.01	0.051
T3-T1	−1.03 (0.92)	−0.45 (0.78)	–2.35	0.046
**TUG-DTc**				
T2-T1	−1.75 (1.22)	−0.28 (1.07)	–4.43	<0.001
T3-T1	−1.67 (1.36)	−0.14 (1.05)	–4.36	<0.001
**MoCA**				
T2-T1	0.58 (0.97)	025 (0.85)	1.265	0.424
T3-T1	0.29 (0.75)	0.38 (0.92)	–0.343	0.733

^$^p-value was adjusted by false discovery rate method. T1, Baseline; T2, postintervention; T3, 4 weeks after the intervention; 6MWT, Six-Minute Walk Test; 30CST, 30-second chair stand test; TUG-DTm, motor dual-task Timed Up-and-Go Test; MoCA, Montreal Cognitive Assessment; LM, Logical Memory of the Wechsler Memory Scale.

## Discussion

To our knowledge, this is the first randomized controlled trial of the effect of Baduanjin on physical and cognitive function with follow-up assessment in middle-aged patients with schizophrenia. We evaluated the effects of Baduanjin exercise and brisk walking matched in duration of intervention. The results showed that both Baduanjin exercise and brisk walking improved physical fitness of the study participants. Badunjin exercise improved global cognition at the end of 12-week intervention; however, the maintenance effect of Baduanjin on global cognition was not significant at follow-up. The Baduanjin group improved significantly more in balance and dual-task performance than the brisk walking group.

With regards to the outcome of physical functions, the Baduanjin and brisk walking group both improved significantly in walking distance and lower extremity muscular strength after the intervention. Our study findings are inconsistent with a previous study that reported no significant effect of Baduanjin in walking distance and lower extremity strength among patients with psychiatric disorders (24). These two studies differed in several aspects. Our study recruited more participants to achieve sufficient statistical power. In addition, the Baduanjin group in our study had more practice sessions for a longer treatment duration than the previous study (24).

A recent study suggested walking speed in schizophrenia was influenced by lower extremity strength (54). The improvements in lower extremity strength in the Baduanjin group in our study might have contributed to the significantly increased walking distance. Our study results were in line with a meta-analytic review that reported evidence for the effects of Baduanjin on walking distance and physical function in older adults (55).

The Baduanjin group in our study improved in the TUG performance after treatment. This finding is in line with a one-group pretest-posttest study which found that Baduanjin improved balance in patients with psychiatric disorders (24). To establish the effects of Baduanjin on balance function in schizophrenia, it is important to include an active control intervention matched in treatment dose for comparison. In our randomized controlled study of Baduanjin vs. brisk walking, the Baduanjin group showed significantly greater improvement in balance than the brisk walking group. This might have been due to the multiple movements required for practice of Baduanjin (e.g., weight shifting, trunk rotation, etc.). Several recent studies have investigated the effects of Baduanjin on balance function in patients with neurologic disease such as stroke (56, 57) and Parkinson disease (58). Continued study is needed of the effects of Baduanjin on ecologically relevant functions of balance; for example, crossing an obstacle in real-life situations.

In our study, the Baduanjin group showed improvement in global cognitive function and logical memory immediately after intervention, but the effect was not maintained at the follow-up assessment. There is growing evidence (15, 16) for the effects of aerobic exercise on cognitive function in patients with schizophrenia. In a recent randomized controlled trial, participants with chronic schizophrenia who practiced traditional Chinese Qigong for 12 weeks improved in Mini-Mental State Examination total score after the intervention (59). Another study showed that patients with schizophrenia who practiced 24 weeks of Baduanjin exercise improved in verbal memory after the intervention.

To interpret the reported benefits of Baduanjin and Qigong for cognitive function in schizophrenia, it should be noted that these physical exercises require light to moderate exertion (22, 59) and focus on breath as well as movement. The cognitive demand for such mindful exercises may have contributed to cognitive enhancement such as attention to body posture and movement sequence. Sufficient amount of practice with graded level of difficulty may be important parameters to allow change to occur in mindful exercises.

A recent meta-analysis of the effect of aerobic exercise suggested at least 90 min per week, at least 12 weeks of treatment duration, with supervision of professionals, and in a group format were core features to lead to improvement in global cognition in schizophrenia (60). A meta-analysis of the effects of physical exercise in older adults with mild cognitive impairment suggested that the total duration of practice should be longer than 24 hours to achieve a robust improvement in global cognition (61). This body of literature indicated the need for addressing the dosing issue in physical exercises, such as Baduanjin exercises, when cognitive functions are the goal of intervention.

Of note, the Baduanjin group in our study showed significant improvement in global cognition and logical memory immediately after the intervention, but the gains were not maintained at follow-up. The findings suggest that a maintenance program should be incorporated into the intervention program to facilitate retention of practice grains from immediately after intervention to the follow-up assessment.

Patients with schizophrenia may encounter difficulties when doing two tasks simultaneously (8). In the present study, we explored the possible benefit of Baduanjin exercise on dual-task performance. Our results showed that the Baduanjin group improved significantly in cognitive dual-task performance. At the follow-up assessment, the Baduanjin group improved in both cognitive and motor dual-task performance. Previous research reported that mind-body exercise, such Tai Chi, might have a positive effect on dual-task performance in older adults (62–64). In line with previous research on older adults, our study revealed that Baduanjin might improve dual-task performance in middle-aged patients with schizophrenia. This effect might be possibly due to the emphasis of Baduanjin on the symmetry of movement and breath and the focus on movement and posture, leading to more cognitive demand than brisk walking.

In our study, Baduanjin and brisk walking both improved mobility and lower extremity strength. Of note, the Baduanjin group improved in cognitive dual-task performance but not motor dual-task performance immediately after the intervention. Improvement in motor dual-task performance was observed at the follow-up assessment. A possible explanation for the delayed effect of Baduanjin on motor dual-task performance pertains to the level of difficulty of the motor dual-task activity (e.g., greater interferences in the motor dual- task condition that required simultaneous processing of breathing, movement acts, and body posture). The findings should be interpreted with caution because of the preliminary nature of the study.

As a limitation to this study, the participants were recruited from a single study site. The findings may not be generalized to other practice settings. Further multi-site research with a larger sample is needed to validate findings of our study. In addition, the dosing issue warrants consideration (e.g., shorter vs. longer duration of intervention). More comprehensive cognitive measures which include different domains of cognition are also needed. A maintenance package may be included to facilitate retention of intervention gains over time. Participant characteristics may moderate treatment response and should be studied to inform patient selection.

## Conclusion

Baduanjin and brisk walking showed comparable effects in walking distance and lower extremity strength in this study. There is a trend for Baduanjin to improve balance and dual-task performance more than brisk walking. The Baduanjin group showed positive effects on global cognition and logical memory immediately after the intervention. Further research with a larger sample from multiple sites is needed to validate findings of the study.

## Data availability statement

The raw data supporting the conclusions of this article will be made available by the authors, without undue reservation.

## Ethics statement

The studies involving human participants were reviewed and approved by the Chang Gung Medical Foundation Institutional Review Board. The patients/participants provided their written informed consent to participate in this study. Written informed consent was obtained from the individual(s) for the publication of any potentially identifiable images or data included in this article.

## Author contributions

C-RC, K-cL, and Y-CH designed the study and developed the treatment programs. C-RC and Y-CL drafted the manuscript. K-cL provided critical review and revisions to the draft. Y-WL and H-HH contributed to the data collection. All authors approved the final manuscript.

## Footnotes

1
www.randomizer.org
